# Pharmacological characterization of potent and selective Na_V_1.7 inhibitors engineered from *Chilobrachys jingzhao* tarantula venom peptide JzTx-V

**DOI:** 10.1371/journal.pone.0196791

**Published:** 2018-05-03

**Authors:** Bryan D. Moyer, Justin K. Murray, Joseph Ligutti, Kristin Andrews, Philippe Favreau, John B. Jordan, Josie H. Lee, Dong Liu, Jason Long, Kelvin Sham, Licheng Shi, Reto Stöcklin, Bin Wu, Ruoyuan Yin, Violeta Yu, Anruo Zou, Kaustav Biswas, Les P. Miranda

**Affiliations:** 1 Neuroscience, Amgen Discovery Research, Thousand Oaks, California, United States of America; 2 Therapeutic Discovery, Amgen Discovery Research, Thousand Oaks, California, United States of America; 3 Molecular Engineering, Amgen Discovery Research, Cambridge, Massachusetts, United States of America; 4 Atheris Laboratories, CH Bernex, Geneva, Switzerland; 5 Discovery Attribute Sciences, Amgen Discovery Research, Thousand Oaks, California, United States of America; 6 Neuroscience, Amgen Discovery Research, Cambridge, Massachusetts, United States of America; Xuzhou Medical University, CHINA

## Abstract

Identification of voltage-gated sodium channel Na_V_1.7 inhibitors for chronic pain therapeutic development is an area of vigorous pursuit. In an effort to identify more potent leads compared to our previously reported GpTx-1 peptide series, electrophysiology screening of fractionated tarantula venom discovered the Na_V_1.7 inhibitory peptide JzTx-V from the Chinese earth tiger tarantula *Chilobrachys jingzhao*. The parent peptide displayed nominal selectivity over the skeletal muscle Na_V_1.4 channel. Attribute-based positional scan analoging identified a key Ile28Glu mutation that improved Na_V_1.4 selectivity over 100-fold, and further optimization yielded the potent and selective peptide leads AM-8145 and AM-0422. NMR analyses revealed that the Ile28Glu substitution changed peptide conformation, pointing to a structural rationale for the selectivity gains. AM-8145 and AM-0422 as well as GpTx-1 and HwTx-IV competed for ProTx-II binding in HEK293 cells expressing human Na_V_1.7, suggesting that these Na_V_1.7 inhibitory peptides interact with a similar binding site. AM-8145 potently blocked native tetrodotoxin-sensitive (TTX-S) channels in mouse dorsal root ganglia (DRG) neurons, exhibited 30- to 120-fold selectivity over other human TTX-S channels and exhibited over 1,000-fold selectivity over other human tetrodotoxin-resistant (TTX-R) channels. Leveraging Na_V_1.7-Na_V_1.5 chimeras containing various voltage-sensor and pore regions, AM-8145 mapped to the second voltage-sensor domain of Na_V_1.7. AM-0422, but not the inactive peptide analog AM-8374, dose-dependently blocked capsaicin-induced DRG neuron action potential firing using a multi-electrode array readout and mechanically-induced C-fiber spiking in a saphenous skin-nerve preparation. Collectively, AM-8145 and AM-0422 represent potent, new engineered Na_V_1.7 inhibitory peptides derived from the JzTx-V scaffold with improved Na_V_ selectivity and biological activity in blocking action potential firing in both DRG neurons and C-fibers.

## Introduction

Toxin peptides derived from venoms have provided key insights into ion channel pharmacology, structure and function in normal and pathophysiological disease states [1]. Voltage-gated sodium channels (Na_V_), essential for the initiation and propagation of action potentials in excitable cells, represent a rich target family for toxin peptides that both inhibit and activate Na_V_ function [2–5]. Spider venoms are especially enriched in Na_V_ modulators with one-third of spider toxin peptides targeting Nav channels pharmacologically [6]. A single spider venom can contain more than 1,000 bioactive peptides [7] with only 0.01% of the over 10 million bioactive spider peptides biologically characterized [6, 8], highlighting the importance of studying spider venom peptides to identify both Na_V_ tools and potential therapeutics.

Na_V_1.7 represents a compelling target for the development of both small and large molecule chronic pain clinical candidates due to its exquisite human genetic validation [9–11]. Congenital insensitivity to pain is due to loss-of-function mutations in Na_V_1.7 and results in loss of pain [12, 13], whereas primary erythromelalgia, paroxysmal extreme pain disorder, and small fiber neuropathy are due to gain-of-function mutations in Na_V_1.7 and result in ongoing spontaneous pain [14–16]. In addition, the human congenital insensitivity to pain phenotype is mimicked in Na_V_1.7 knockout mice with cell-type specific knockouts highlighting the involvement of Na_V_1.7 in both inflammatory and neuropathic pain endpoints [17–19]. Development of specific Na_V_1.7 inhibitors as pre-clinical tools and clinical candidates requires inherent selectivity over other Na_V_ isoforms, including Na_V_1.4 natively expressed in skeletal muscle and Na_V_1.5 natively expressed in cardiac myocytes [20]. Molecular selectivity has been achieved by targeting binding sites that are less conserved within the Nav family, in particular the voltage-sensing domains instead of the pore region [10]. Indeed, known Na_V_1.7 inhibitory peptides map to voltage-sensor domains and can reduce sensitivity of channel opening to depolarizing voltage stimuli [3].

Previously, we reported the identification, optimization, and derivatization of the 34 amino acid Na_V_1.7 inhibitory peptide GpTx-1, from the Chilean tarantula *Grammostola porteri*, discovering analogs with single-digit nM Na_V_1.7 IC_50_ values [21–24]. To identify peptides with improved Na_V_1.7 inhibitory potency compared to GpTx-1 analogs, we embarked on another fractionated tarantula venom electrophysiology screen and identified the 29-residue inhibitory cystine knot (ICK) peptide JzTx-V (β/κ-theraphotoxin-Cg2a also known as β-theraphotoxin-Cj2a) with sub-nM Na_V_1.7 IC_50_ from the Chinese earth tiger tarantula *Chilobrachys jingzhao*. JzTx-V was originally described as an inhibitor of native Na_V_ channels in rat DRG neurons with no selectivity over Na_V_1.4 in HEK293 cells [25, 26]. Given the lack of high-resolution co-crystal structures of Na_V_ channels with inhibitory peptides to enable rational design of selectivity, we applied the attribute-based positional scan analoging strategy previously employed for K_V_1.3 and Na_V_1.7 inhibition [22, 27] to engineer selectivity-enhancing JzTx-V modifications that retain Na_V_1.7 potency. This effort led to the discovery of a key Ile28Glu mutation that imparted >100-fold selectivity for Na_V_1.7 over Na_V_1.4. NMR-structure guided analog design and chemical synthesis subsequently yielded the Ile28Glu-containing lead peptides AM-8145 and AM-0422. Here we report on the *in vitro* and ex vivo pharmacological characterization of these engineered peptides, including specific block of rodent action potential firing in DRG neurons and C-fibers following capsaicin and mechanical stimulation compared to an inactive peptide AM-8374 devoid of these biological activities.

## Materials and methods

### Isolation and purification of JxTx-V

Venom from the tarantula *C*. *jingzhao* (also known as *Chilobrachys guangxiensis*) was extracted via electrical stimulation of an anesthetized spider (Atheris Laboratories, Switzerland, Melusine ref. MLU_020110). Venom samples were collected, lyophilized, and dissolved in 0.1% trifluoroacetic acid (TFA) in water to approximately 1 mg venom/mL. The crude venom solutions were desalted by solid-phase extraction (SPE) with Sep-Pak C18 cartridges (Waters, Milford, MA, USA) equilibrated in 0.1% TFA and eluted with 60% aqueous acetonitrile and then evaporated. The dried material was dissolved in 0.1% TFA to approximately 1 mg venom/mL concentration, and higher molecular weight components were removed with a 10 kDa molecular weight cut-off Ultrafree-CL centrifugal filter (Millipore). The <10 kDa venom extract was then dried under vacuum and stored at -80°C.

The crude venom was fractionated by reversed phase (RP)-HPLC. Less than 10 kDa venom extracts were dissolved in 0.1% TFA to approximately 1mg venom/mL, separated by C18 RP-HPLC chromatography (Phenomenex Jupiter 5 μm C18 300 Å 250 x 2.0 mm; detection at 214 nm), and collected into 84 samples corresponding to approximately 1 minute wide fractions. HPLC method: Buffer A (0.1% TFA in water) and buffer B (90% acetonitrile/10% water containing 0.1% TFA) at 1 mL/min with a 1% /min gradient 0–100% buffer B. The fractions were transferred into a plate format, dried under vacuum, and stored at -80°C.

The venom fractions were screened for activity in a Na_V_1.7 IonWorks Quattro (IWQ) assay. Several fractions with significant (>50% of control) Nav1.7 inhibitory activity were identified. A second aliquot of the fractions was tested against Na_V_1.7, Na_V_1.4, and Na_V_1.5 IWQ assays to confirm the activity of the initial hit and evaluate selectivity. The most potent and selective fraction, fraction 36, was analyzed by matrix-assisted laser desorption ionization time-of-flight mass spectrometry (MALDI-TOF MS) using an α-cyano-4-hydroxycinnamic acid matrix. The concentration of peptide in this fraction was not determined. The major signal indicated a molecular weight (3602.34 Da) consistent with the known Na_V_ inhibitory peptide toxin JzTx-V (3602.65 Da, UniProt Accession Number Q2PAY4) [25]. To confirm the identity and biological activity of JzTx-V, the peptide was chemically synthesized and tested.

### Peptide synthesis, oxidative folding, and purification

Rink Amide Chem Matrix resin (0.2 mmol, 0.45 mmol/g loading, 0.444 g, Matrix Innovation) was weighed into a CS BIO reaction vessel. The reaction vessel was connected to a channel of the CS BIO 336X automated peptide synthesizer, and the resin was washed twice with DMF and allowed to swell in DMF for 15 min. Fmoc-amino acid (1.0 mmol, Midwest Biotech or Novabiochem) was dissolved in 2.5 mL of 0.4 M 6-chloro-1-hydroxybenzotriazole (6-Cl-HOBt, Matrix Innovation) in DMF. To the solution was added 1.0 mL of 1.0 M 1,3-diisopropylcarbodiimide (DIC, Sigma-Aldrich) in DMF. The solution was agitated with nitrogen bubbling for 15 min to accomplish pre-activation and then added to the resin. After the mixture was shaken for 2 h, the resin was filtered and washed 3 x DMF, 2 x DCM, and 3 x DMF. Fmoc-removal was accomplished by treatment with 20% piperdine in DMF (5 mL, 2 x 15 min, Fluka). The resin was filtered and washed 3 x DMF. All residues were single coupled through repetition of the Fmoc-amino acid coupling and Fmoc removal steps described above.

After final Fmoc-removal from the N-terminal residue, resin-bound linear peptide (0.2 mmol scale) was transferred to a 25 mL SPE filter tube, washed 3 x DMF and 3 x DCM, and dried under vacuum. To the resin were added triisopropylsilane (1.0 mL), 3,6-dioxa-1,8-octane-dithiol (DODT, 1.0 mL), water (1.0 mL), TFA (15 mL), and a stir bar, and the mixture was stirred for 3 h. The mixture was filtered into a 50 mL centrifuge tube. The resin was washed with TFA (~ 5 mL), and the combined filtrate was concentrated by rotary evaporation in a Genevac HT-12 (30°C chamber temperature, pressure ramp from 500 to 50 mbar over 40 min and a final pressure of 8 mbar for 2 h). To the residue (~5 mL) was added 40 mL cold diethyl ether, leading to the formation of a white precipitate. The white ether suspension was stirred and centrifuged (4 min, 4,400 rpm), and the ether was decanted. To the tube was added another 40 mL of cold ether, and the precipitate was stirred. The mixture was centrifuged, and the ether was decanted. The solid was dried overnight under vacuum. The crude linear peptide was purified by preparative LC-MS. The filtered sample (300 mg in 5 mL DMSO with 0.1 M tris(2-carboxyethyl)phosphine hydrochloride (TCEP, Sigma-Aldrich)) was injected onto a preparative HPLC column (Phenomenex Synergi 4um MAX-RP 80A AXIA, 250 x 30 mm). The peptide was eluted with a 10–40%B over 60 min gradient at 30 mL/min, followed by a 10 min flush and a 10 min equilibration. The fractions were analyzed by LC-MS, pooled, and lyophilized to afford the pure linear peptide precursor.

In a 1L polypropylene bottle was prepared a folding buffer with water (800 mL), acetonitrile (100 mL), cysteine (1 mL of a 1 M stock solution in water), and cystine dihydrochloride (6.667 mL of a 150 mM stock solution in water). To the pure linear peptide (100 mg) was added 5 mL acetonitrile and 5 mL water. The mixture was vortexed and sonicated to complete dissolution of the peptide. The peptide solution was added to the buffer followed by 1M Tris-HCl pH 8.0 (100 mL), (0.1 mg/mL peptide concentration, 1 mM cysteine, 1 mM cystine, 10% v/v acetonitrile, 0.1 M Tris pH 8.0). The pH value was measured to be 8.0. The folding mixture was allowed to stand at 4°C for 18 to 72 h. A small aliquot was removed, and the sample was analyzed by LC-MS to ensure that the folding was complete. The solution was quenched by the addition of 4 mL acetic acid (HOAc) and 4 mL TFA (pH = 2.5). The aqueous solution was filtered (0.45 μM cellulose membrane).

The filtered solution (1000 mL, 100 mg peptide) was loaded onto a preparative HPLC column (Phenomenex Synergi 4um MAX-RP 80A AXIA, 250 x 30 mm) at 30 mL/min using an Agilent preparative loading pump. The column was flushed for 10 min with 10% B at 30 mL/min to elute the HOAc/TFA. The column was attached to a preparative HPLC, Agilent/LEAP prep LC-MS, and the peptide was eluted with a 10–40% B gradient over 60 min, followed by a 10 min flush and a 10 min equilibration. The fractions were analyzed by LC-MS, pooled, and lyophilized to afford pure folded peptide.

Final LC-MS analysis (Phenomenex Jupiter 20 × 2 mm, 100 Å, 5 micron column eluted with a 10 to 60% B over 10 min gradient at a 0.750 mL/min flow rate monitoring absorbance at 220 nm) was performed. Peptides with > 95% purity and correct (m/z) ratio were screened.

### NMR structural analysis

The structures of peptides 3 and AM-8145 were obtained by high resolution NMR spectroscopy using Bruker Avance III 600 MHz and 500 MHz NMR systems both utilizing a TCI cryoprobe (Billerica, MA). Samples were prepared in 20 mM sodium phosphate, pH 7.0 containing 50 mM NaCl and 10% D_2_O. The temperature of all NMR spectra were optimized (320K for peptide 3 and 325K for AM-8145) to minimize effects of exchange broadening and facilitate signal resolution.

The assignment of chemical shifts correlating to the backbone and side-chain (all protons and Cα/Cβ only) resonances of both peptides were determined using standard methods from a combination of the two-dimensional NOESY, TOCSY, HSQC, and HMBC spectra. NOE-based distance restraints were determined from NOESY spectra utilizing mixing times of 500 ms. Additional NOEs were obtained from the ω^1^-decoupled TOCSY-NOESY experiment that provided dipolar correlations between cysteine residues [28]. These restraints were incorporated manually. Stereospecific proton assignments were obtained based on the coupling patterns between C′-Hβ and Hα-Hβ evaluated from ^13^C-HSQC and HMBC experiments [29]. Dihedral angle constraints for the χ_1_ angles (N-Cα-Cβ-S) were determined, when possible, using the NOE patterns between Hα-Hβ and Hα-Hβ′, along with the stereospecific assignments and ^3^**J**_HαHβ_ and ^3^**J**_HαHβ′_ coupling constants measured from the high resolution NOESY or TOCSY spectra.

Structure determination for both peptides proceeded by manually assigning NOEs using standard methods. The NOE assignments and structure calculations were carried out from extended strand starting structures using Cyana 3.0 [30] and performed iteratively along with interactive assignment by the user. In each of seven rounds, 100 structures were calculated using 50,000 steps of torsion angle dynamics. Once structures had begun to converge, hydrogen bond restraints were incorporated manually. At the final stage, disulfide bond constraints were added according to the determined connectivity patterns. χ_1_ angle constraints were added based on ranges determined using the Karplus equation [31] and were incorporated with relatively loose restraints (60 ± 30°). Structures were re-calculated as above, and the 10 structures with the lowest target functions were selected for analysis.

### Radioligand binding assay

^125^I-ProTx-II was purchased from PerkinElmer Life Sciences with a specific activity of 2,200 Ci/mmol. HEK293 cells stably expressing human Na_V_1.7 were seeded at 75,000 cells/well in 96-well poly-D-lysine-coated plates (Corning) and allowed to attach for 16–20 h at 37°C. Prior to assay, cell culture medium was replaced with assay buffer containing DMEM/F12 (Gibco) with 0.1% fetal bovine serum (Gibco). For saturation binding experiments, cells were incubated with increasing concentrations of ^125^I-ProTx-II for 3 hours and then washed twice with 200 μl of assay buffer. 100 μl of liquid scintillation fluid (Microscint-40, PerkinElmer Life Sciences) was added, and radioactivity was counted using a TopCount^TM^ microplate scintillation counter (PerkinElmer). Triplicate samples were averaged for each experimental data point. Total binding was defined as counts in the presence of ^125^I-ProTx-II alone, nonspecific binding was defined as counts remaining when ^125^I-ProTx-II was co-incubated with cold 1 μM AM-8145, and specific binding was obtained by subtracting nonspecific binding from total binding counts. K_d_ and B_max_ values were calculated using Graphpad Prism 7 (GraphPad Software Inc.). For competition binding experiments, 0.5 nM ^125^I-ProTx-II was incubated with cells for 3 h in the absence or presence of increasing concentrations of ProTx-II, HwTx-IV, GpTx-1 and JzTx-V peptides. Inhibition constant (K_i_) values were calculated using GraphPad Prism 7.

### Electrophysiology

Human Na_V_ (hNa_V_) stably transfected cell lines in HEK293 or CHO backgrounds and cell culture methods were as previously described [32]. hNa_V_1.1-hNa_V_1.7 cells were obtained from Eurofins Pharma Discovery Services (St. Charles, MO), and hNa_V_1.8 cells were generated in house. Whole cell patch clamp, PatchXpress, and IWQ electrophysiology voltage protocols as well as DRG neuron isolation were carried out as previously described [24]. Na_V_1.7-Na_V_1.5 chimeras were generated and evaluated in BacMam transduced U2-OS cells on the IWQ platform as previously described [23].

### Multi-electrode array (MEA)

Action potentials of cultured postnatal day 2 rat DRG neurons (Lonza, Walkersville, MD) were detected using the Maestro multi-well plate-based MEA system (Axion Biosystems, Atlanta, GA). A 48-well MEA plate containing 16 electrodes/well was pre-coated with 10μL 0.1% PEI (Sigma-Aldrich, St. Louis, MO) in borate buffer (50mM boric acid (Fisher Scientific, Waltham, MA), 12mM sodium tetraborate (Sigma-Aldrich), pH 8.4). The plate was incubated at 37°C for 2h, washed 3-times with 200μL sterile ddH_2_O and then air dried. Next, 10μL of 20μg/mL laminin (Sigma) in Primary Neural Basal Medium (PNBM) (Lonza) was spotted over the PEI-treated electrode surface and incubated for 2h at 37°C. Laminin was removed and immediately replaced with 10μL/well of 7x10^6^ cells/mL rat DRGs in PNBM. After 1h at 37°C, 300μL of PNBM (supplemented with 2 mM L-glutamine (Lonza), 1% gentamycin/amphotericin (Lonza), 2% NSF-1 (Lonza), 100 ng/mL NGF-β (Sigma-Aldrich), and an additional 50mM NaCl to yield a final NaCl concentration of ~127mM) was added and cells were maintained at 37°C, 5% CO_2_ for a total of 8–9 days, with a fresh media exchange after 4 days. Compound inhibition of capsaicin-induced action potential firing was measured at 37°C. Baseline spontaneous firing was recorded for approximately 1 min. Twenty-five μL of PNBM, 325nM capsaicin (MP Biomedicals, 25nM final, EC_80_) in PNBM, or 325nM capsaicin plus test compound was then added, and action potentials were continuously recorded for another 2 min with six replicate wells used per condition. MEA data were analyzed using Axion Integrated Studio AxIS2.1 (Axion Biosystems) and GraphPad Prism version 7.02 for Windows (GraphPad Software, La Jolla, CA).

### Ethics statement

Experimental procedures were approved by Amgen Inc.’s Institutional Animal Care and Use Committee in accordance with the National Institutes of Health's Guide for the Care and Use of Laboratory Animals and conducted at an AALAC-accredited facility.

### Skin-saphenous nerve preparation

Male C57BL/6 mice (Charles River Laboratories, San Diego, CA) between 2–3 months old (136 total mice used in study) were housed under standard temperature conditions (22–24°C) and illumination (12-hour light/dark cycle with lights on at 6:30 AM) with ad libitum access to fresh water and food. Mice were euthanized by CO_2_ followed by cervical dislocation. Experimental procedures for mouse skin-saphenous nerve studies were conducted as previously described with the use of 0.1% BSA (Acros) in the bath solution to decrease non-specific loss of peptide to chamber components [19] [32]. Briefly, the saphenous nerve and skin from the left medial dorsum of the hind paw were dissected and mounted in a custom-made organ bath chamber. The proximal saphenous nerve end was passed through a hole into a separate recording chamber, desheathed and teased into fine fibers for extracellular recording. Mechanical responses were evoked by square force waves using a mechanical stimulator before and following peptide application to a small area of the corium (dermis) side of the skin in a custom-made stainless steel ring. Recording and analysis of C-fiber-mediated action potentials were performed in a blinded manner with respect to peptide treatment.

## Results

### Screening venom fractions to identify JzTx-V

**Fig 1:**
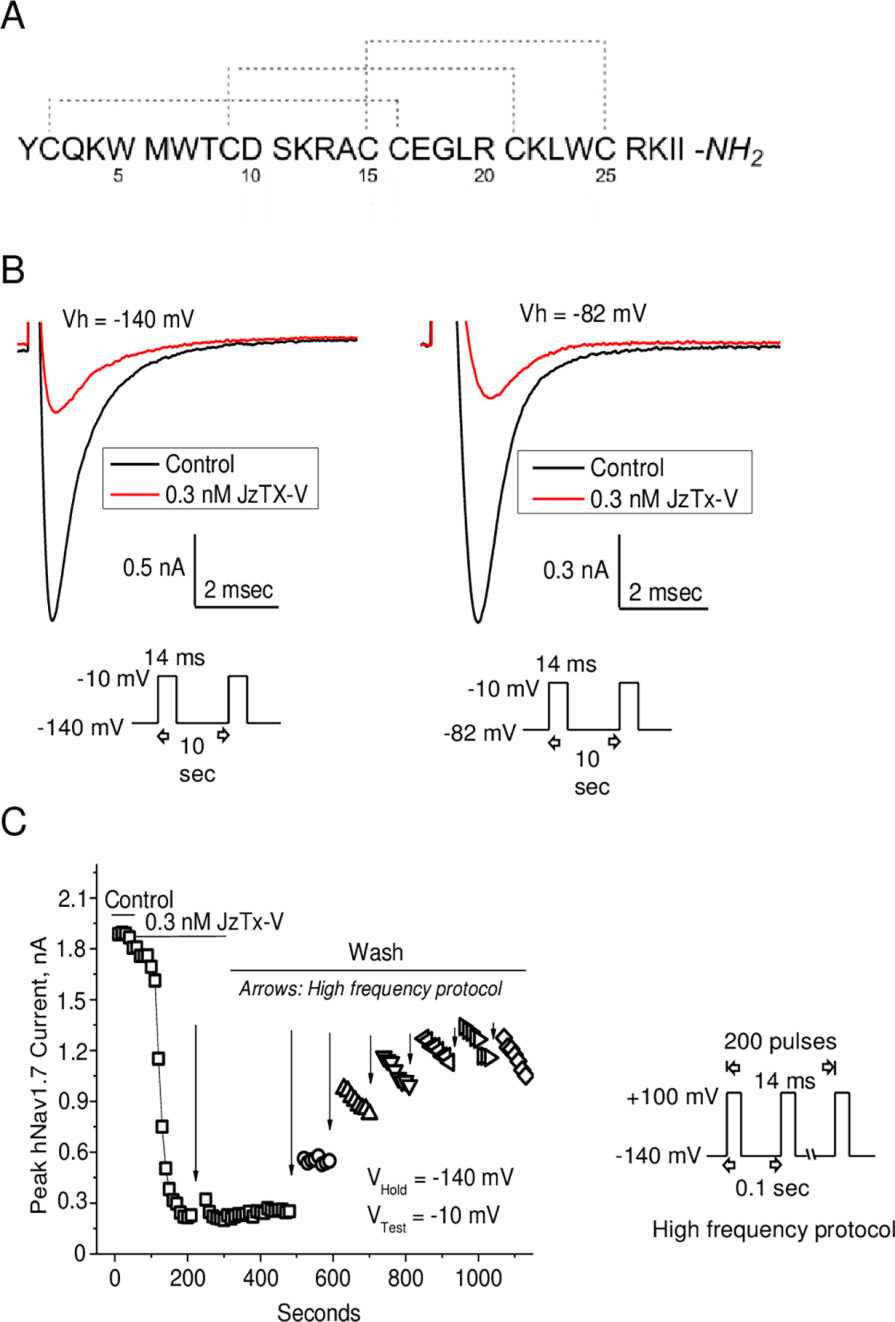
JzTx-V sequence and inhibition of Na_V_1.7 currents in HEK293 cells. A. Amino acid sequence and disulfide connectivity of JzTx-V. B. Manual patch clamp traces for control (black) and JzTx-V (0.3 nM; red) channel block at a holding potential of -140 mV (left) or -82 mV (right). Voltage protocols are depicted below the traces. C. JzTx-V (0.3 nM) channel block is partially reversed by high-frequency strong depolarizations following peptide washout. Cells were held at -140 mV and stepped to -10 mV to record Na_V_1.7 current. Downward arrows indicate time points during which a high frequency protocol (depicted to right of time course; step to +100 mV for 14msec at 10 Hz for 20 sec) was applied.

Our previous experience with GpTx-1 had shown spider venoms from the Theraphosidae family were enriched in Na_V_ inhibitory peptides [24]. We acquired fractionated venoms from a number of tarantula species to add to our natural peptide collection and screened them for Na_V_1.7 inhibitory activity with the intention of identifying a more potent starting lead molecule. Over eighty percent inhibition of Na_V_1.7 current was observed for fraction 36 from the Chinese earth tiger tarantula *C*. *jingzhao* (S1 Fig). MALDI-TOF analysis revealed a single major pattern that contained the bioactive component identified as Jingzhaotoxin-V (JzTx-V, UniProtKB–Q2PAY4; S2 Fig). JzTx-V is a 29 amino acid polypeptide with a C-terminal amide and 6 cysteine residues engaged in 3 disulfide bonds to form an ICK motif and is a member of NaSpTx family 3 (Fig 1A) [6].

### Potency and selectivity of JzTx-V

JzTx-V was originally described as a non-selective inhibitor of Na_V_ channels in rat DRG neurons [25]. Synthetic JzTx-V (HPLC profile shown in S3 Fig) was evaluated against human Na_V_1.7 heterologously expressed in HEK293 cells on a PatchXpress automated electrophysiology platform, using a voltage protocol in which 20% of channels were in the inactivated state, and yielded an IC_50_ of 0.63 ± 0.17 nM (n = 4). The potency of JzTx-V against Na_V_1.4 revealed 3- to 4-fold selectivity over Na_V_1.7 (Na_V_1.4 IC_50_ = 2.2 ± 0.4 nM, n = 3), and the potency of JzTx-V against Na_V_1.5 revealed nearly 4,000-fold selectivity over hNa_V_1.7 (Na_V_1.5 IC_50_ = 2,350 ± 480 nM, n = 3). Manual patch clamp electrophysiology studies were conducted to evaluate the mechanism of action for JzTx-V channel blockade of hNa_V_1.7. The potency of JzTx-V inhibition of Na_V_1.7 was 0.15 ± 0.05 nM (n = 2) by manual patch, using the same voltage protocol as above; this value is slightly lower than obtained on the PatchXpress platform and likely due to improved cell perfusion. JzTx-V inhibition of hNa_V_1.7 in the resting/closed state (0.3 nM JzTx-V blocked 83 ± 2% current at a holding potential of -140 mV) or a partially-inactivated state (0.3 nM JzTx-V blocked 83 ± 6% current at a holding potential of -80 mV) was comparable, indicating peptide block was not state-dependent across these voltages and proceeded via interaction with a closed state (Fig 1B). High frequency strong depolarizations to +100 mV partially reversed JzTx-V block of Na_V_1.7, indicating lower peptide affinity for the channel open state(s) and displacement of the peptide from its binding pocket upon the closed to open gating state transition (Fig 1C).

### Na_V_ isoform selectivity engineering to discover AM-8145 and AM-0422

**Fig 2:**
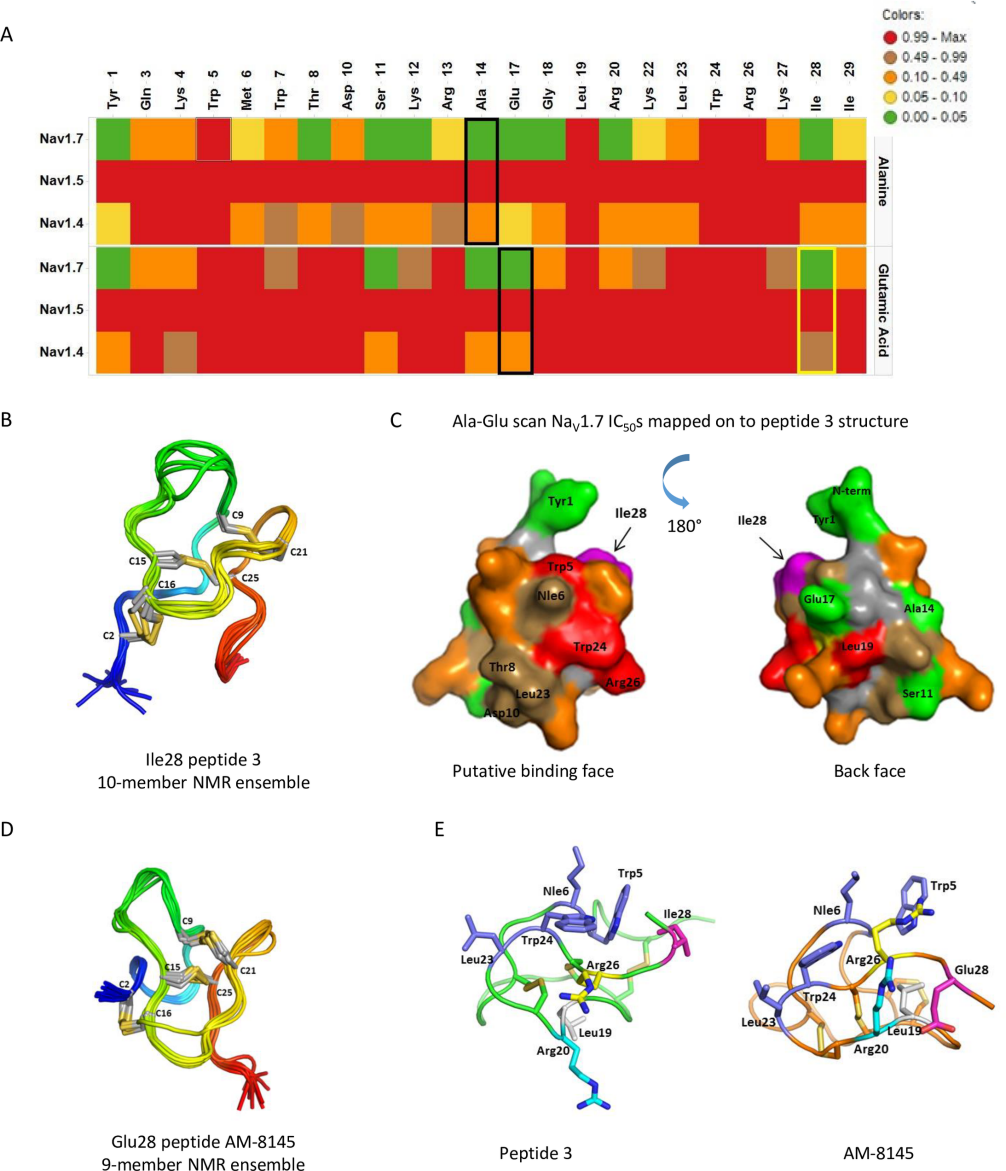
Ala/Glu scan heat map and NMR structure of JzTx-V peptides. A. Heat map showing single residue scan IC_50_ data of Ala- and Glu-mutants against Na_V_1.7, Na_V_1.5 and Na_V_1.4 using the IWQ platform. Black rectangles indicated wild-type JzTx-V sequences and the yellow rectangle indicates the Ile28 mutation that confers selectivity to Na_V_1.4. Cys residues were not mutated. B. NMR structural ensemble of peptide 3 containing the Ile28 residue. Disulfides are in yellow, the N-terminal region in blue and the C-terminal region in red. C. Average of Ala- and Glu-mutant Na_V_1.7 IC_50_’s mapped onto a space-filling model of peptide 3 based on the NMR structure, showing key residues that lose Na_V_1.7 activity upon modification cluster on one face of the peptide. Colors correspond to IC_50_ ranges in panel A. Residues tolerant to modification are in green and located on the reverse side of the putative binding face. The Ile28 residue is colored magenta. D. NMR structural ensemble of peptide AM-8145 containing the Glu28 residue that imparts selectivity over Na_V_1.4. Colors are as in panel B. E. Comparison of key amino acid side chains from peptide 3 and AM-8145 NMR structures. The Glu28 modification induced a change in overall conformation, with the acidic Glu28 side chain pointing down towards the basic Arg20 in AM-8145.

Since the selectivity of native JzTx-V for Na_V_1.7 over Na_V_1.4 was only 3–4 fold, we set out to improve Na_V_1.4 isoform selectivity by the single residue mutation attribute-based positional scanning paradigm we previously described [22]. Alanine scanning mutagenesis of all non-cysteine residues via chemical synthesis and refolding was performed and the resulting peptides were tested against Na_V_1.7, Na_V_1.4 and Na_V_1.5 using the IWQ platform. The resulting IC_50_ data identified key residues for Na_V_1.7 block, exemplified by Trp5, Leu19, Trp24 and Arg26 (Fig 2A, S1 Table). Similar to the parental JzTx-V peptide, Ala-mutants did not block Na_V_1.5 function. However, none of the Ala-mutants conferred significant selectivity over Na_V_1.4. Attribute-based positional scanning of tarantula toxin GpTx-1 showed maximum disruption of Na_V_ activity with the negatively charged glutamic acid residue [22]. Therefore, we prepared and tested Glu-mutants of JzTx-V as above. The Na_V_1.7 IC_50_ data showed Met6, Thr8, Asp10, Arg13 and Leu23 were additionally involved in the interaction with Na_V_1.7 (Fig 2A, S1 Table). Interestingly, Glu-scanning mutagenesis revealed a significant advance in generating selective Na_V_1.7 inhibitors from the JzTx-V scaffold in the form of the Ile28Glu mutation that showed good selectivity over Na_V_1.4. Peptide 1, [Glu28]JzTx-V(1–29), potently blocked Na_V_1.7 (IC_50_ = 0.6 nM), was 500-fold selective against Na_V_1.4 (IC_50_ = 301 nM) and was a weak blocker of Na_V_1.5 (IC_50_ = 8,800 nM) on the PX platform.

We next sought to identify suitable locations on the JzTx-V sequence where we could attach handles for potential derivatization by incorporating an alkyne side-chain containing residue in the sequence, e.g. propargylglycine (Pra) [23]. The Glu-scan data suggested specific positions where such mutations would have minimum impact on Na_V_1.7 potency (exemplified by Glu-scan analog Na_V_1.7 IC_50_’s < 50 nM, represented in green in Fig 2A). The N-terminus, positions 1, 11, 14 and 17 were selected for the incorporation of Pra. For this study, we replaced Met6 with the isosteric residue norleucine, to avoid complications from side-chain sulfide oxidation during synthesis and folding. The Pra analogs of [Glu28]JzTx-V(1–29) were tested against Na_V_1.7, Na_V_1.4 and Na_V_1.5 (Table 1). Adding a Pra-residue to the N-terminus gave peptide 2 (AM-8145), which was equipotent to WT JzTx-V and demonstrated excellent selectivity over Na_V_1.4 (300-fold) and Na_V_1.5 (6,000-fold). For an exact comparison, the peptide was remade without the key selectivity-determining Glu28 residue. Peptide 3, which has the native Ile residue at position 28 and thus differs from AM-8145 by only one amino acid, was only 5-fold selective against Na_V_1.4, replicating the dramatic selectivity difference we initially observed between WT JzTx-V and the IleGlu28 analog. Varying the Pra location to positions 1, 11 and 14 while retaining Glu28 gave peptides 4–6 with similar potent inhibition of Na_V_1.7 and selectivity against Na_V_1.4 (200–300 fold). The Pra17 analog 7 was 6-fold less potent compared to WT JzTx-V and AM-8145. Interestingly, when a N-terminal residue with a side chain isosteric to the alkyne in AM-8145, β-cyanoalanine CyA, was added to 7, the resulting peptide 8 (AM-0422) displayed similar potency to AM-8145 (Na_V_1.7 IC_50_ = 0.8 nM) on the PX platform.

**Table 1:** Potency and selectivity of JzTx-V and peptide analogs.

Peptide	Peptide Name	Na_V_1.7 IC_50_ (nM)	Na_V_1.4 IC_50_ (nM)	Na_V_1.5 IC_50_ (nM)
JzTx-V	JzTx-V(1–29)	0.6	2.2	2,350
1	[Glu28]JzTx-V(1–29)	0.6	301	8,800
2 (AM-8145)	Pra-[Nle6;Glu28]JzTx-V(1–29)	0.5	145	3,000
3	Pra-[Nle6]JzTx-V(1–29)	1.2	6.2	1,087
4	[Pra1;Nle6;Glu28]JzTx-V(1–29)	0.3	89	4,600
5	[Nle6;Pra11;Glu28]JzTx-V(1–29)	0.5	194	6,000
6	[Nle6;Pra14;Glu28]JzTx-V(1–29)	0.5	110	3,500
7	[Nle6;Pra17;Glu28]JzTx-V(1–29)	3.6	130	3,100
8 (AM-0422)	CyA-[Nle6;Pra17;Glu28]JzTx-V(1–29)	0.8	103	966
9 (AM-8394)	Pra-[Nle6;Glu19,28]JzTx-V(1–29)	>1000	n.d.	n.d.

IC_50_ values of JzTx-V analogs on human Na_V_1.7, Na_V_1.4 and Na_V_1.5 as determined on the PX platform. IC_50_ values were determined from at least ten different cells with two to three data points per peptide concentration as previously described [24]. n.d. = not determined. All peptides contain C-terminal amides.

### JzTx-V analog NMR structure

The NMR structures of JzTx-V peptide analogs were determined with and without the Na_V_1.4 selectivity-conferring Glu28 residue to evaluate the impact of this modification on peptide conformation. The structure of peptide 3, Pra-[Nle6]JzTx-V(1–29) (PDB 6CHC), containing the wild-type Ile28 residue was determined at 320K (RMSD for residues 5–30 was 0.45 ± 0.07Å) and confirmed the ICK folded motif with disulfide connectivity identified between Cys2-Cys16, Cys9-Cys21 and Cys15-Cys-25 (Fig 2B). Super-imposing averaged Ala- and Glu-scan analog heat map data onto the NMR structure of peptide 3 revealed residues with the greatest loss in Na_V_1.7 potency were clustered on one face of the folded peptide, the purported ion channel binding face (Fig 2C). The back or reverse face of the peptide is comprised of residues shown to be replaceable without compromising Na_V_1.7 inhibition, and appear to be reasonable locations for Pra insertions for further derivatization.

The NMR structure of the Glu28 analog of peptide 3, AM-8145 (PDB 6CGW), was solved at 325K (RMSD of 0.71 ± 0.14 Å, Fig 2D). The signals from residues Lys12-Arg13-Ala14 were broadened suggesting local disorder and thus complete assignment of that region was not possible. The Ile28Glu modification caused a change in the structure of the C-terminal region of the peptide (Fig 2E). The acidic Glu28 side chain is reoriented towards the basic Arg20 functionality. In the AM-8145 structure, Leu19 packs into the core of the peptide adjacent to Trp5 and towards the purported ion channel binding face. We also observed a modest reorientation of the sidechains of Trp24 and Arg26 within the mapped binding surface. Residues critical for activity continue to cluster on one face of the peptide; though the overall shape of the presumed binding face is changed. Significant changes in the N-terminal conformation and Asp10-Ala14 loop were also observed; however, these changes were in a region of the AM-8145 peptide that showed local disorder and was tolerant to substitution, as mutation to Ala or Pra had minimal effect on activity (peptides 5–7). The conformational change induced by the Ile28Glu substitution and subtle alteration of the presumed binding face may help rationalize the Nav1.4 selectivity gains achieved with this amino acid change.

### JzTx-V binding to Na_V_1.7 cells

**Fig 3:**
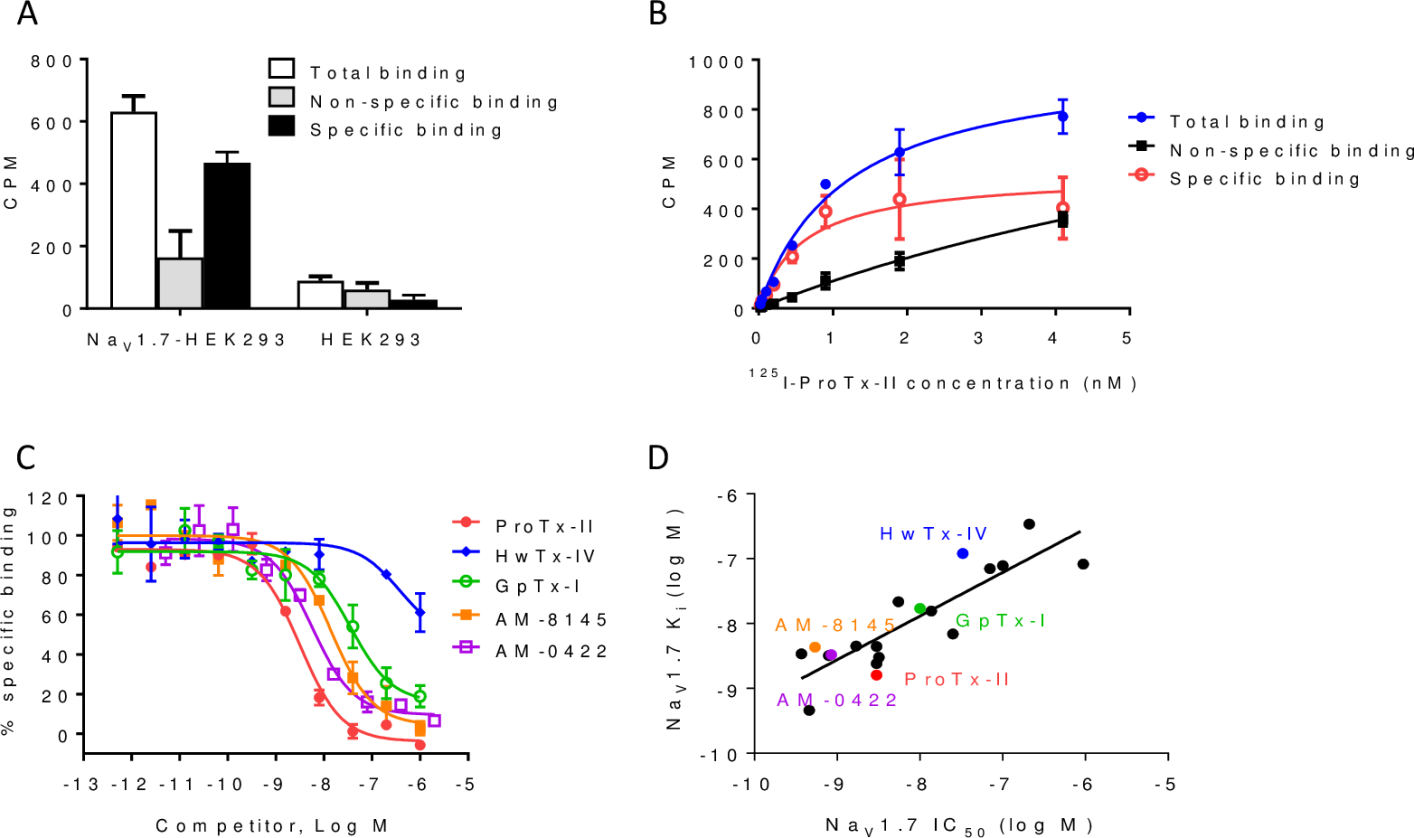
JzTx-V peptides displace ^125^I-ProTx-II binding to hNa_V_1.7 HEK293 cells. A. Binding of ^125^I-ProTx-II (1.5 nM) to Na_V_1.7 HEK293 but not parental cells. Specific binding counts were displaced by cold AM-8145 (1 μM). B. Saturation curves for ^125^I-ProTx-II binding. Closed circles (blue) represent total binding, filled squares (black) represent non-specific binding, and open circles (red) represent specific binding, defined as the difference between total and non-specific binding. C. Competition binding curves with 0.5 nM ^125^I-ProTx-II in the presence of increasing concentrations of the indicated peptides. ^125^I-ProTx-II binding is competed by AM-8145, AM-0422, GpTx-1 and ProTx-II peptides. D. Correlation of Na_V_1.7 IC_50_ values by PX electrophysiology (x-axis) with K_i_ values for binding (y-axis) for a panel of reference and JzTx-V peptides (R^2^ of 0.74).

Na_V_1.7 inhibitory peptide binding to live HEK293 cells stably expressing Na_V_1.7 was evaluated. Using the radiolabeled peptide ^125^I-ProTx-II and cold peptide AM-8145 (1 μM), specific interaction with HEK293-Na_V_1.7 but not parental HEK293 cells was observed (Fig 3A). ^125^I-ProTx-II binding had a K_d_ of 0.61 ± 0.2 nM (n = 3) and B_max_ of 540 ± 62.8 (2,700 sites per cell) in saturation binding experiments (Fig 3B). Using this assay, a panel of cold Na_V_1.7 inhibitory peptides was evaluated, including the tarantula venom peptides ProTx-II, HwTx-IV and GpTx-1. These diverse peptides all competed for ^125^I-ProTx-II binding, indicating interaction with the same Na_V_1.7 binding site (Fig 3C). Extension of this panel to encompass a collection of JzTx-V peptide analogs, including AM-0422 and AM-8145, revealed a similar rank order between binding (K_i_) and functional block by electrophysiology on the PatchXpress platform (IC_50_) (Fig 3D). Collectively, these data demonstrate that JzTx-V peptides specifically bind to Na_V_1.7 channels and that peptide binding reflects channel block as assessed by electrophysiology.

### AM-8145 chimera mapping to the Na_V_1.7 second voltage-sensor domain

**Fig 4:**
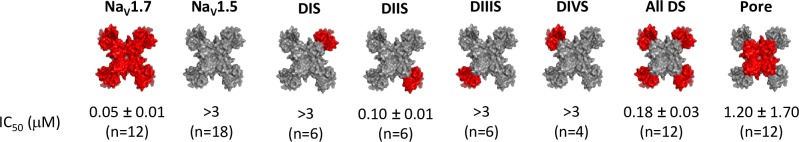
AM-8145 maps to the second voltage sensor domain on hNa_V_1.7. Potency of AM-8145 inhibition of wild-type Na_V_1.7, wild-type Na_V_1.5, or chimeric Na_V_1.7/Na_V_1.5 channels (red illustrates Na_V_1.7 sequence and gray illustrates Na_V_1.5 sequence) on the IWQ platform (mean ± SEM). D = “Domain”; S = “Sensor” corresponding to transmembrane segments 1–4; P = “Pore” corresponding to transmembrane segments 5–6. AM-8145 block of wild-type Na_V_1.7 is similar to chimera DIIS containing the second voltage sensor domain of Na_V_1.7 embedded in the Na_V_1.5 backbone.

Experiments were conducted to map the binding site of JzTx-V peptide AM-8145 on Na_V_1.7. Given the lack of robust bioactivity of AM-8145 on Na_V_1.5, we evaluated AM-8145 functional block on a battery of human Na_V_1.7-Na_V_1.5 chimeric channels, in which individual voltage sensor domains (transmembrane domains S1-S4 of domains I-IV) or the entire pore domain (transmembrane domains S5-S6 of domains I-IV) of Na_V_1.7 were inserted into the Na_V_1.5 backbone. As shown in Fig 4, AM-8145 potently blocked wild-type Na_V_1.7 (IC_50_ = 0.05 ± 0.01 μM) but not wild-type Na_V_1.5 (IC_50_ > 3 μM) when evaluated on the IWQ platform. Chimeras with all four Na_V_1.7 voltage-sensor domains (IC_50_ = 0.18 ± 0.03 μM) or only the second Na_V_1.7 voltage-sensor domain (IC_50_ = 0.10 ± 0.01 μM) showed potent channel block by AM-8145 similar to wild-type Na_V_1.7, whereas chimeras with only the first, third, or fourth Na_V_1.7 voltage-sensor domain did not exhibit potent block (IC_50_ > 3 μM). A chimera with the Na_V_1.7 pore domain exhibited weak block by AM-8145 with an IC_50_ value that was right-shifted over 20-fold (IC_50_ = 1.2 ± 1.7 μM) compared to wild-type Na_V_1.7. Taken together, these data indicate that AM-8145 interacts with the second voltage-sensor domain of Na_V_1.7.

### AM-8145 Na_V_ isoform selectivity

**Fig 5:**
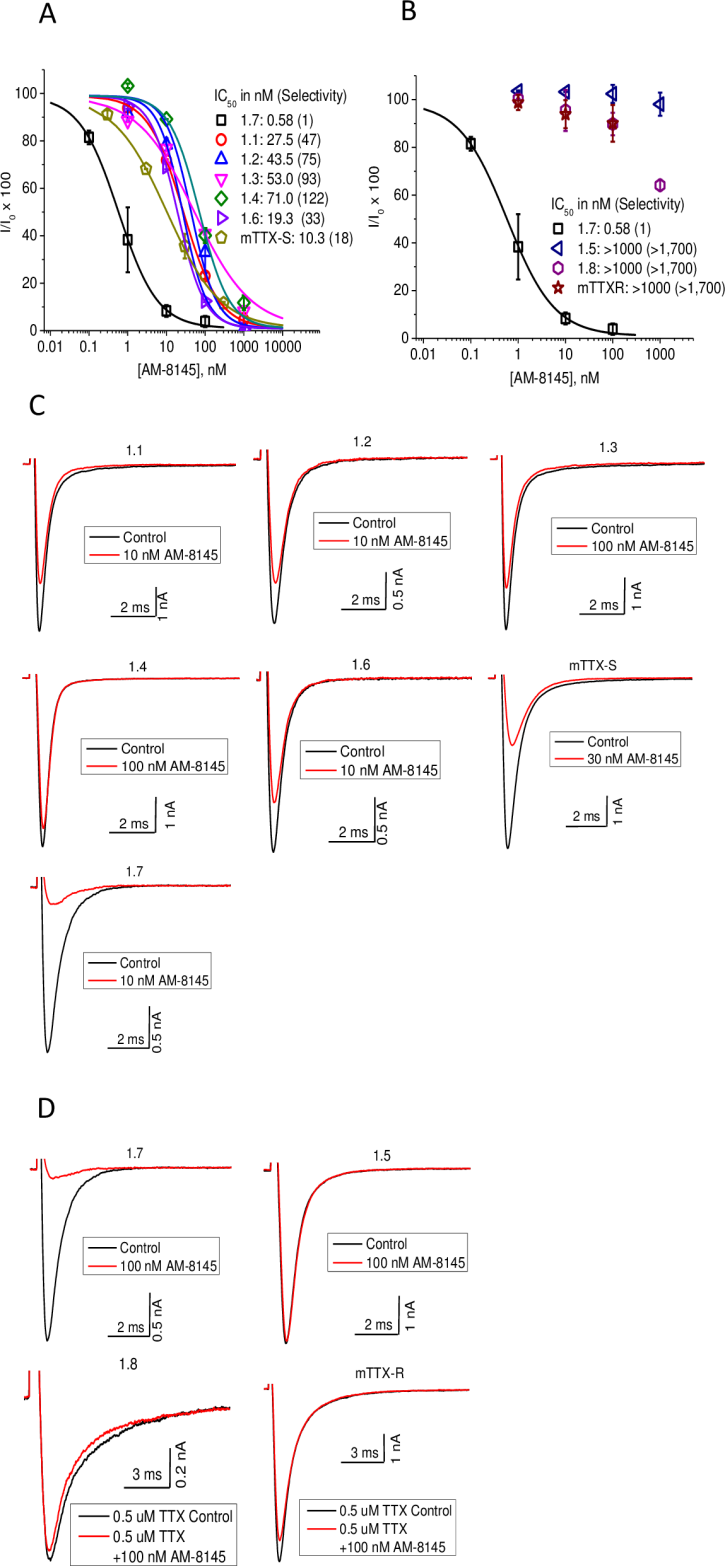
Selectivity of AM-8145 for human Na_V_ channels in heterologous cells and native Na_V_ currents in mDRG neurons. A. Concentration response curves for TTX-S channels including Na_V_1.1-Na_V_1.4, Na_V_1.6-Na_V_1.7 and mDRG TTX-S. IC_50_ values with fold-selectivity compared to Na_V_1.7 are listed in parenthesis. AM-8145 was 30- to 120-fold selective for Na_V_1.7 over Na_V_1.1-Na_V_1.4 and Na_V_1.6. B. Concentration response curves for TTX-R channels including Na_V_1.5, Na_V_1.8 and mDRG TTX-R. AM-8145 was over 1,700-fold selective for Na_V_1.7 over Na_V_1.5, Na_V_1.8 and mDRG TTX-R. Curves in A and B were derived from whole cell patch clamp experiments. Data are presented as mean ± SEM with n = 2–4 cells per data point. C. Representative current traces for TTX-S channels before (black) and after (red) 10 nM AM-8145 addition. Cells were held at a voltage yielding 20% channel inactivation and stepped to -10 mV for 14 msec. D. Representative current traces for TTX-R channels before (black) and after (red) 100 nM AM-8145 addition. Cells were held at a voltage yielding 20% channel inactivation and stepped to -10 mV for 14 msec (Na_V_1.5), or 0 mV for 15msec (Na_V_1.8 and mDRG TTX-R). Studies with mDRG TTX-R included 0.5 uM TTX to block endogenous TTX-S currents. 10 nM AM-8145 preferentially inhibited Na_V_1.7.

The potency and selectivity of AM-8145 on human Na_V_ isoforms (Na_V_1.1-Na_V_1.8) as well as native Na_V_ channels sensitive to tetrodotoxin (TTX-S) or resistant to tetrodotoxin (TTX-R) in mouse DRG neurons were evaluated using a manual patch clamp electrophysiology voltage protocol in which 20% of Na_V_ channels were inactivated. AM-8145 exhibited an IC_50_ of 0.58 nM on Na_V_1.7 and was 30- to 120-fold selective over other human TTX-S Nav channels including Na_V_1.1, Na_V_1.2, Na_V_1.3, Na_V_1.4, and Na_V_1.6 (Fig 5A and 5C). AM-8145 potently inhibited native TTX-S channels, largely comprised of native Na_V_1.7 [33], in mouse DRG neurons with an IC_50_ of 10.3 nM. AM-8145 was over 1,000-fold selective over human TTX-R channels including Na_V_1.5 and Na_V_1.8 as well as native TTX-R Na_V_1.8 channels in mouse DRG neurons with IC_50_ values > 1 μM (Fig 5B and 5D). In summary, AM-8145 potently blocks human Na_V_1.7 and native TTX-S channels in mouse DRG neurons, exhibits 30- to 120-fold selectivity over other human TTX-S channels, and exhibits over 1,000-fold selectivity over other human TTX-R channels.

### AM-0422 and less active analog AM-8394 potency on rodent DRG neurons

**Fig 6:**
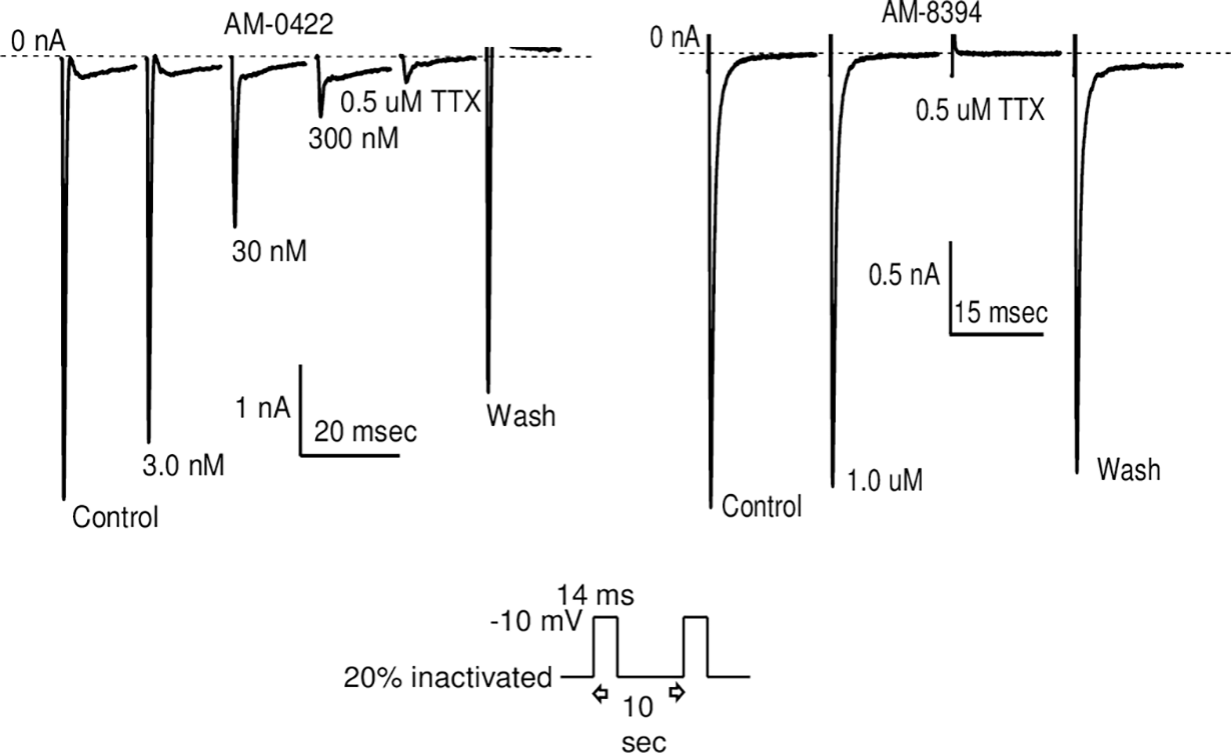
**Representative whole cell patch clamp recordings for active peptide AM-0422 (left) and inactive peptide AM-8394 (right) showing reversible block of TTX-S Na**_**V**_ **channels in mDRG neurons.** TTX was used as a positive control. AM-0422 IC_50_ 27.6 ± 7.7 nM (n = 4), and AM-8394 was inactive up to 1 uM (3.5±1.1% inhibition, n = 2). Voltage protocol is depicted below the traces.

To further evaluate the pharmacology of JzTx-V peptides in biological assays measuring action potential firing in rodent DRG neurons or peripheral nerve fibers, we generated a pair of structurally similar peptides with disparate bioactivities. Since the Na_V_1.7 potency and selectivity over Na_V_1.4 and Na_V_1.5 were comparable for AM-8145 and AM-0422, which mainly differ in positioning of the Pra residue for future derivatization, we selected AM-0422 as the active peptide for these experiments. AM-0422 potently blocked native TTX-S Na_V_ channels in mouse (IC_50_ 27.6 ± 7.7 nM, n = 4) and rat (IC_50_ 9.3 ± 1.3 nM, n = 2) DRG neurons whereas AM-8145 peptide analog AM-8394 (Table 1), containing a Leu19Glu modification, Pra-[Nle6;Glu19,28]JzTx-V(1–29), was inactive at the highest concentration tested (1 μM AM-8394 caused 3.5 ± 1.1% inhibition), when assessed by manual patch clamp whole cell electrophysiology using a voltage protocol where 20% of Na_V_ channels were inactivated (Fig 6). AM-8394 was designed and synthesized for this study based on the loss in Na_V_1.7 potency when the wild-type Leu19 residue was replaced by either Ala or Glu during scanning experiments (Fig 2A). When evaluated against human Na_V_1.7 and mouse Na_V_1.7 channels in heterologous HEK293 cells, AM-8394 did not potently block sodium currents at the highest concentration tested (1 μM). Thus, AM-8394 represents a Ile28Glu containing JzTx-V analog that can be used as a negative control peptide in pharmacology studies.

### AM-0422 and less active analog AM-8394 inhibition of action potential firing in rat DRG neurons by MEA

**Fig 7:**
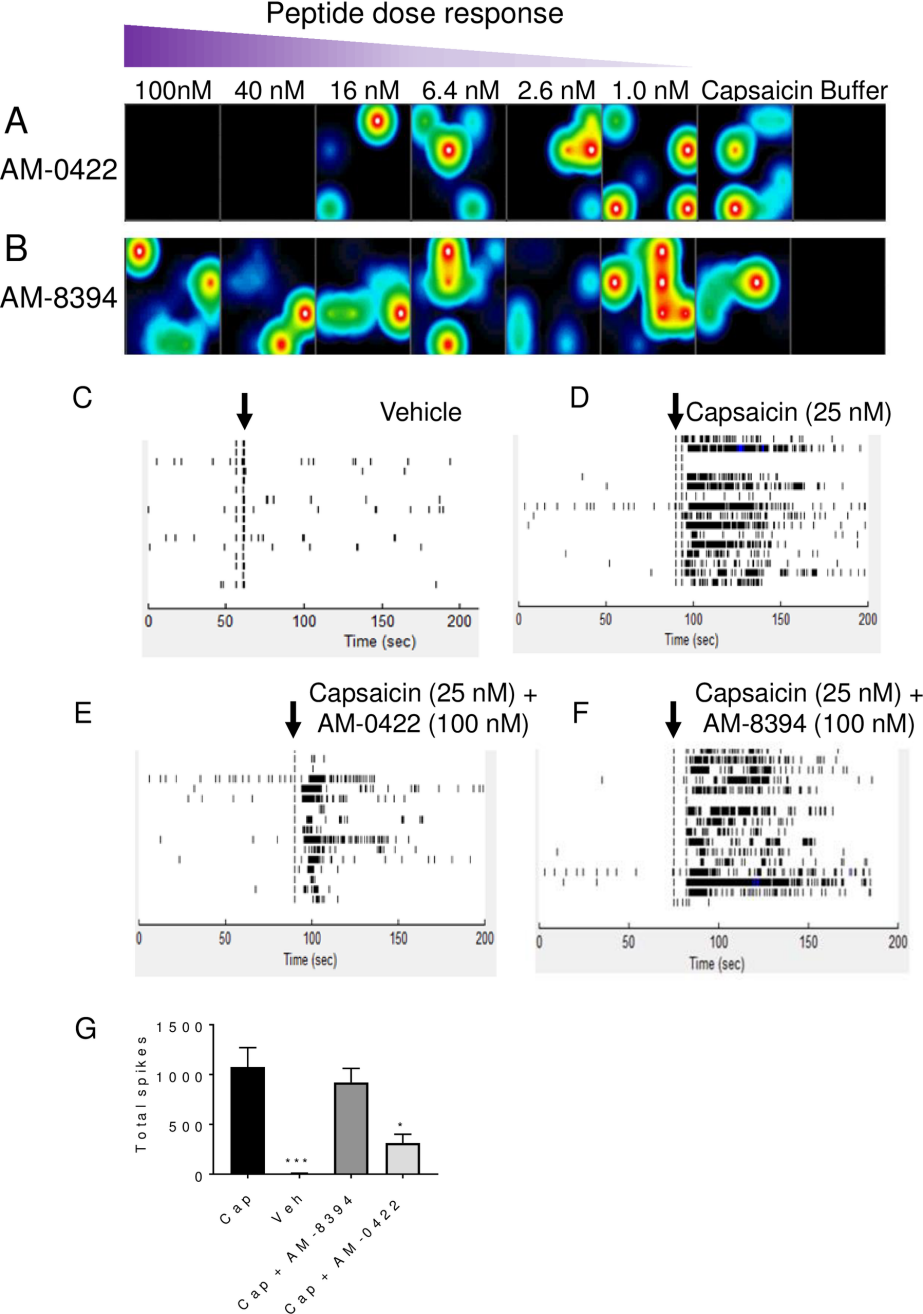
Inhibition of capsaicin-induced action potential firing in MEA recordings in rat DRG neurons by active peptide AM-0422 but not inactive peptide AM-8374. Heat maps of active electrodes detecting action potential firing 30 seconds following vehicle, 25 nM capsaicin, 25 nM capsaicin plus 1–100 nM of AM-0422 (A) or 25 nM capsaicin plus 1–100 nM of AM-8394 (B). The colors white/red, yellow/green, and blue/black correspond to high, medium, and low action potential firing frequency respectively. Whole well raster plots for buffer (C), 25 nM capsaicin (D), 25 nM capsaicin plus 100 nM AM-0422 (E), or 25 nM capsaicin plus 100 nM AM-8394 (F) added at the time point indicated by the vertical black arrows. Each row in the raster plots represents a single electrode with 16 electrodes per well and all recordings were performed at 37°C. G. Summary of total spikes from active wells following 2 minute treatment with 25 nM capsaicin (Cap), vehicle, 25 nM capsaicin plus 100 nM AM-8394 or 25 nM capsaicin plus 100 nM AM-0422. *** p<0.001, * p<0.05 by two-tailed unpaired t-test compared to capsaicin group.

Experiments were performed to evaluate the ability of AM-0422 to block action potential firing in response to the nociceptive agent capsaicin in neurons that natively express Na_V_1.7 channels. Using cultured rat DRG neurons grown on a multi-electrode array, 25 nM capsaicin (corresponding to an EC_80_ concentration), but not buffer control, increased the frequency of action potential firing for a period of two minutes following application (Fig 7A, 7C, 7D and 7G). When co-applied with capsaicin, the active peptide AM-0422, but not the less active peptide AM-8394, dose-dependently decreased capsaicin-induced action potential firing (Fig 7A, 7B and 7E–7G). The IC_50_ for AM-0422 block was 21.1 ± 5.7 nM whereas the IC_50_ for AM-8394 was >1 μM. Taken together, these data indicate that AM-0422 specifically blocks capsaicin-induced rat DRG neuron action potential firing.

### AM-0422 and less active analog AM-8394 inhibition of action potential firing in skin-saphenous nerve assay

**Fig 8:**
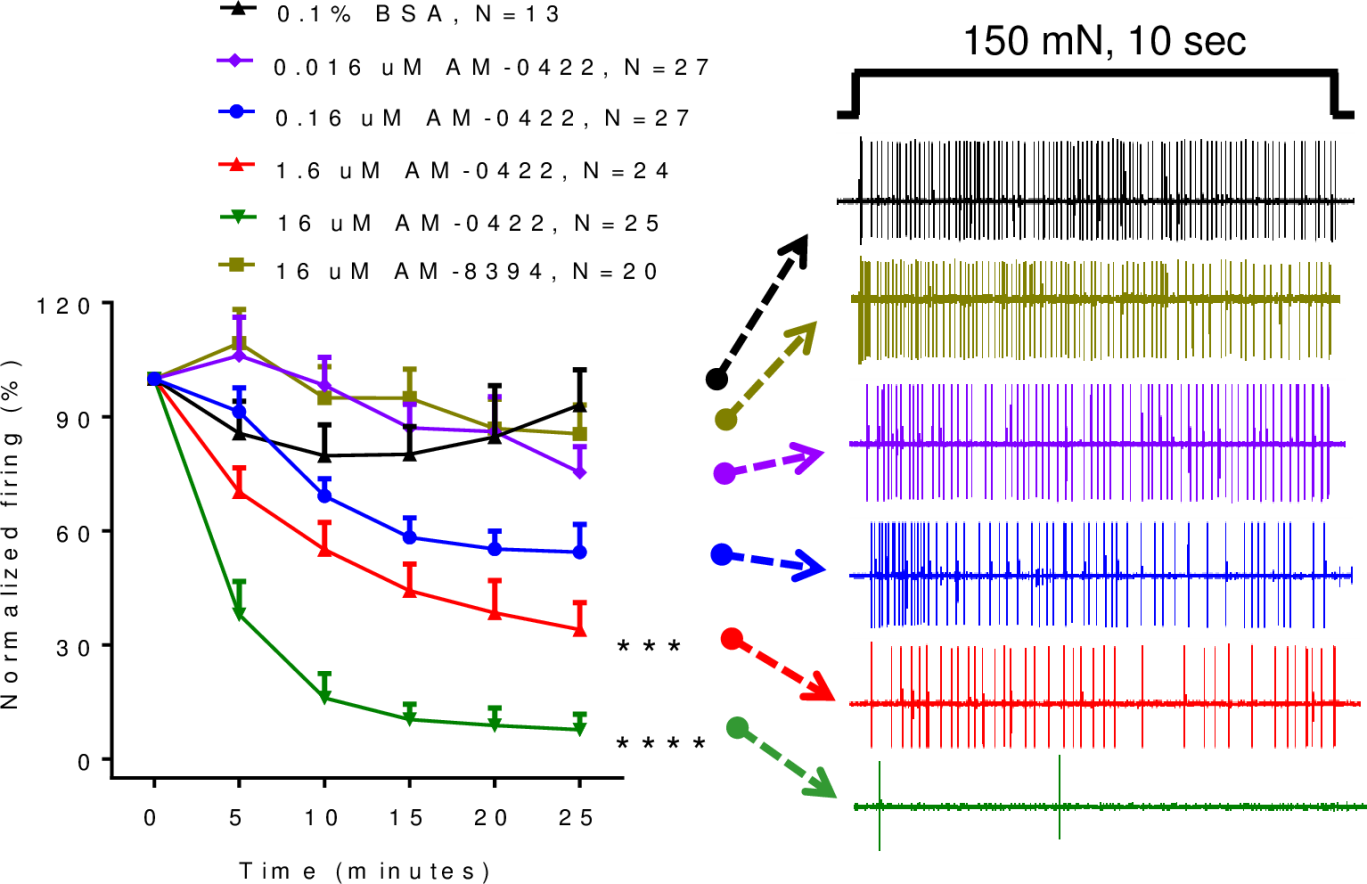
Inhibition of mechanically-evoked action potential firing in TTX-sensitive C-fibers by active peptide AM-0422 but not inactive peptide AM-8374. Time- and concentration-dependent inhibition of action potential firing by AM-0422. *** P < 0.001, **** P = 0.0001 by repeated measure two-way ANOVA with Dunnett’s multiple comparison test compared to 0.1% BSA vehicle. Representative traces for each group are shown for the 25 min time point on the right. 150 mN mechanical stimuli were applied for 10 sec every 5 min. Data are mean ± SEM; n = 13–27 fibers per group.

The mouse skin-saphenous nerve tissue preparation represents an *ex vivo* experimental system to evaluate action potential firing in nerve fibers that originate from peripheral receptive fields in the skin and are transmitted along the saphenous nerve. We previously demonstrated that both TTX and a Na_V_1.7-specific small molecule sulfonamide gating modifier were able to inhibit mechanically-induced C-fiber action potential firing in this preparation [32]. Therefore, experiments were performed to evaluate the impact of AM-0422 in this system. When locally applied to a receptive field on the skin, AM-0422 dose-dependently blocked spiking in response to a 150 mN mechanical stimulus compared to 0.1% BSA vehicle control (Fig 8; repeated measures two-way ANOVA, F(5,130) = 34, P = <0.0001) with an IC_50_ of 880 nM. When evaluated at 16 μM, the highest concentration of AM-0422 tested, the less active peptide AM-8394 had no effect on spiking. Taken together, these data indicate that AM-0422 specifically blocks mechanically-induced C-fiber action potential firing from afferent nerve terminals.

## Discussion

AM-8145 and AM-0422 represent potent and selective engineered JzTx-V-derived peptide inhibitors of recombinant human Na_V_1.7 channels in heterologous cells and native TTX-S Na_V_ channels in mouse and rat DRG neurons. AM-0422 inhibited capsaicin-induced action potential firing in rat DRG neurons and mechanically-induced C-fiber action potential firing in a mouse skin-nerve preparation. The inactive control peptide AM-8394 did not block action potential firing, pointing to a specific requirement for Na_V_ target engagement in these endpoints. This represents the first report characterizing the pharmacology of an active and inactive Na_V_1.7 peptide pair in a collection of *in vitro* and *ex vivo* experiments.

JzTx-V was originally described as an inhibitor of both TTX-S (IC_50_ = 28 nM) and TTX-R (IC_50_ = 30 nM) sodium channels in rat DRG neurons with additional inhibitory activity against the voltage-gated potassium channels K_V_4.1, K_V_4.2 and K_V_2.1 [25] [34]. Consistent with our findings, JzTx-V exhibited weak inhibition of Na_V_1.5 and had nominal selectivity over Na_V_1.4 when evaluated in heterologous HEK293 cells [26]. In order to identify a better compound for further interrogation of Na_V_1.7 biology and encouraged by the inherent potency of the parent JzTx-V peptide, we sought to engineer improved selectivity against Na_V_1.4 using the JzTx-V scaffold. Given the lack of structural information of human Na_V_ channels at the time of this work from X-ray crystallography or cryo-electron microscopy needed to elucidate atomic-level structural differences between the homologous TTX-S sodium channels Na_V_1.7 and Na_V_1.4, we focused on an empirical attribute-based positional scan analoging strategy previously successfully employed for K_V_1.3 and Na_V_1.7 [22, 27]. The effect of a JzTx-V Ala-scan on rat Na_V_1.4 block has been reported [26], and our Ala-mutant data validated the importance of residues Trp5, Trp24 and Arg26 in peptide block of human TTX-S channels. However, we did not observe any indication of differential peptide activity against Na_V_1.7 and Na_V_1.4 with Ala scan mutants. In our previous GpTx-1 engineering study, scanning with the negatively charged glutamic acid residue had the most disruptive effect on bioactivity [22]. Like GpTx-1 and other tarantula venom-derived Na_V_ inhibitory peptides, JzTx-V is basic in nature and we reasoned that a single residue scan with a carboxyl group-containing side chain could lead to novel peptides with modified Na_V_ activity. Therefore, we synthesized and tested the full panel of Glu-scan mutants. The set of Glu JzTx-V single mutants increased the topographic map of residues that may interact with Na_V_1.7 (e.g. residues Met6, Thr8, Leu23), and revealed a key Ile28Glu mutation with >100-fold selectivity for Na_V_1.7 over Na_V_1.4. This finding is a testament to the power of attribute-based positional analoging in peptide lead discovery and optimization when high resolution structural information is unavailable.

Combining scan analoging with NMR structural data showed that key residues for Na_V_1.7 block were clustered on one face of the folded peptide while modifications on the opposite or back face were more tolerated. We identified locations on the back surface for attaching synthetic handles for future peptide derivatization that retained Na_V_1.7 potency and selectivity. This effort yielded the lead JzTx-V peptides AM-8145 and AM-0422 for further investigation of Na_V_1.7 biology. Interestingly, the Leu19Glu analog of AM-8145 (AM-8394) lost bioactivity, despite the modified residue mapping to the peptide core based on the AM-8145 NMR structure, indicating that the contribution of certain residues in blocking ion channel function may be due to indirect conformational effects. In any case, the outcome afforded access to the inactive JzTx-V mutant AM-8394 as a negative control in biological experiments.

To further understand Nav1.4 selectivity gains of the Ile28Glu substitution, we solved the NMR structures of peptide 3 and its Glu28 analog, AM-8145. The Ile28Glu substitution reorients the C-terminus of the peptide with Glu28 in close proximity to Arg20. This results in the reorientation of Leu19 and Arg26, a subtle shift in the binding face of AM-8145 as well as larger conformational changes on the peptide back face. The change in the geometry and composition of the binding face may contribute to the Nav1.4 selectivity observed with this single residue change. Presently, no high-resolution structure of a mammalian Na_V_ containing the second voltage-sensor domain is available. Given the dynamic nature of Na_V_ gating and the rigid protein-protein docking methods available to us, we did not have sufficient confidence that docking studies would afford the resolution or accuracy necessary to explain the observed Na_V_1.4 selectivity.

JzTx-V functions as a gating modifier peptide toxin that stabilizes Na_V_1.7 in a closed conformation with state-independent block between -140 and -80 mV. JzTx-V has lower affinity for the channel open state(s) and high frequency strong depolarizations that cycle Na_V_1.7 through closed, open, and inactivated conformations partially reversed JzTx-V channel block. Using a ProTx-II radiolabeled cell-based binding assay, a diverse panel of cold Na_V_1.7 inhibitory peptides including JzTx-V derivatives (AM-8145 and AM-0422), GpTx-1, HwTx-IV, and ProTx-II competed for the same binding site with a good correlation between Na_V_1.7 IC_50_ values by electrophysiology and K_i_ values by binding. A panel of Na_V_1.7-Na_V_1.5 chimeras was employed to map AM-8145 to the second voltage-sensor domain of Na_V_1.7. Further Na_V_1.7-Na_V_1.5 chimeras with specific extracellular loop swaps did not narrow Na_V_1.7 regions necessary for peptide block in preliminary experiments. Collectively, the landscape of different tarantula-derived Na_V_1.7 inhibitory peptides including the JzTx-V derivatives described here, ProTx-II, HwTx-IV, GpTx-1 and additional newly described ICK peptides all share similar biophysical mechanisms of action including channel block via gating modulation, lower affinity for channel open states, and mapping to specific channel voltage-sensor domains in particular the second voltage sensor region [23, 35–46].

AM-0422 dose-dependently blocked action potential firing induced by the algogen capsaicin in cultured rat DRG neurons in a MEA assay. Nominal inhibition was observed with the inactive control peptide AM-8394. The half-maximal inhibitory concentration of AM-0422 in the spiking MEA assay was 21 nM, which was two to three-fold higher than the native TTX-S Na_V_ IC_50_ of 9 nM in rat DRG neurons. Similarly, AM-0422 dose-dependently blocked mechanically-induced action potential firing in mouse C-fibers in a skin-nerve preparation assay, and no inhibition was observed with the inactive peptide AM-8394. The half-maximal inhibitory concentration of AM-0422 in the skin nerve assay was 880 nM, which was thirty-fold higher than the native TTX-S Na_V_ IC_50_ in mouse DRG neurons. We were unable to evaluate AM-0422 block of capsaicin-induced action potential firing in the skin nerve preparation due to desensitization of capsaicin responses that precluded repeated testing before and after peptide pharmacological challenge. Higher concentrations of AM-0422 needed to block spiking in nerve fibers in the skin-nerve preparation compared to dissociated DRG neurons in culture could be attributable to dermal barriers that limit peptide access to Nav channels in *ex vivo* tissue systems, as previously described for the Na_V_1.7 small molecule sulfonamide antagonist AMG8379 [32] and the Na_V_ pore blocker TTX [47]. Consistent with this proposal of limited biodistribution to Na_V_ channels, AM-0422 effects required 20–25 minutes of constant peptide perfusion to achieve full block, and the Na_V_1.7 inhibitory peptide ProTx-II only impacted electrically-induced C-fiber action-potential firing following desheathing of the saphenous nerve to enable peptide access [48]. The highest concentration of AM-0422 tested (16 uM) nearly fully inhibited C-fiber action potential firing, whereas both AMG8379 and Nav1.7 KO mice yielded 60 percent C-fiber inhibition [19, 32]. The additional block by AM-0422 may be due to engagement of other TTX-S Nav channels in C-fibers, such as Na_V_1.1 and Na_V_1.6 [49–52].

In summary, using a combination of attribute-based positional scan analoging and NMR structural analyses, we have engineered potent and selective Na_V_1.7 inhibitory peptides derived from the JzTx-V scaffold. AM-8145 and AM-0422 represent research tools with sub-nM potency on human Na_V_1.7 and 100-1000-fold selectivity over Na_V_1.4 and Na_V_1.5 that can be utilized to interrogate Na_V_1.7 biology in cell and tissue-based preparations. Continued peptide optimization efforts to develop analogs that can engage Na_V_1.7 *in vivo* and block pharmacodynamic endpoints are ongoing.

## Supporting information

S1 Table Potency of JzTx-V Ala and Glu scan mutations. IC_50_ values of JzTx-V peptides against human Na_V_1.7, Na_V_1.4, and Na_V_1.5 as determined on the IWQ platform. Wild-type JzTx-V peptide sequence is listed in Column 1. Cysteine residues were not mutated (shaded in green).
(XLSX)

S1 Fig RP-HPLC fractionation of crude venom extracted from *C*. *jingzhao*. (PPTX)

S2 Fig MALDI-TOF MS analysis of fraction 36 from *C*. *jingzhao* venom. (PPTX)

S3 Fig Analytical RP-HPLC trace of synthetic JzTx-V (r_t_ = 4.96 min). (PPTX)
