# Supplementary material for: Pharmacological characterization of potent and selective NaV1.7 inhibitors engineered from Chilobrachys jingzhao tarantula venom peptide JzTx-V
Source: PLoS One. 2018 May 3;13(5):e0196791. doi: 10.1371/journal.pone.0196791 (PMC5933747; doi:10.1371/journal.pone.0196791)
Supplement: S1 Fig — (PPTX) [file pone.0196791.s002.pptx]

## Slide 1
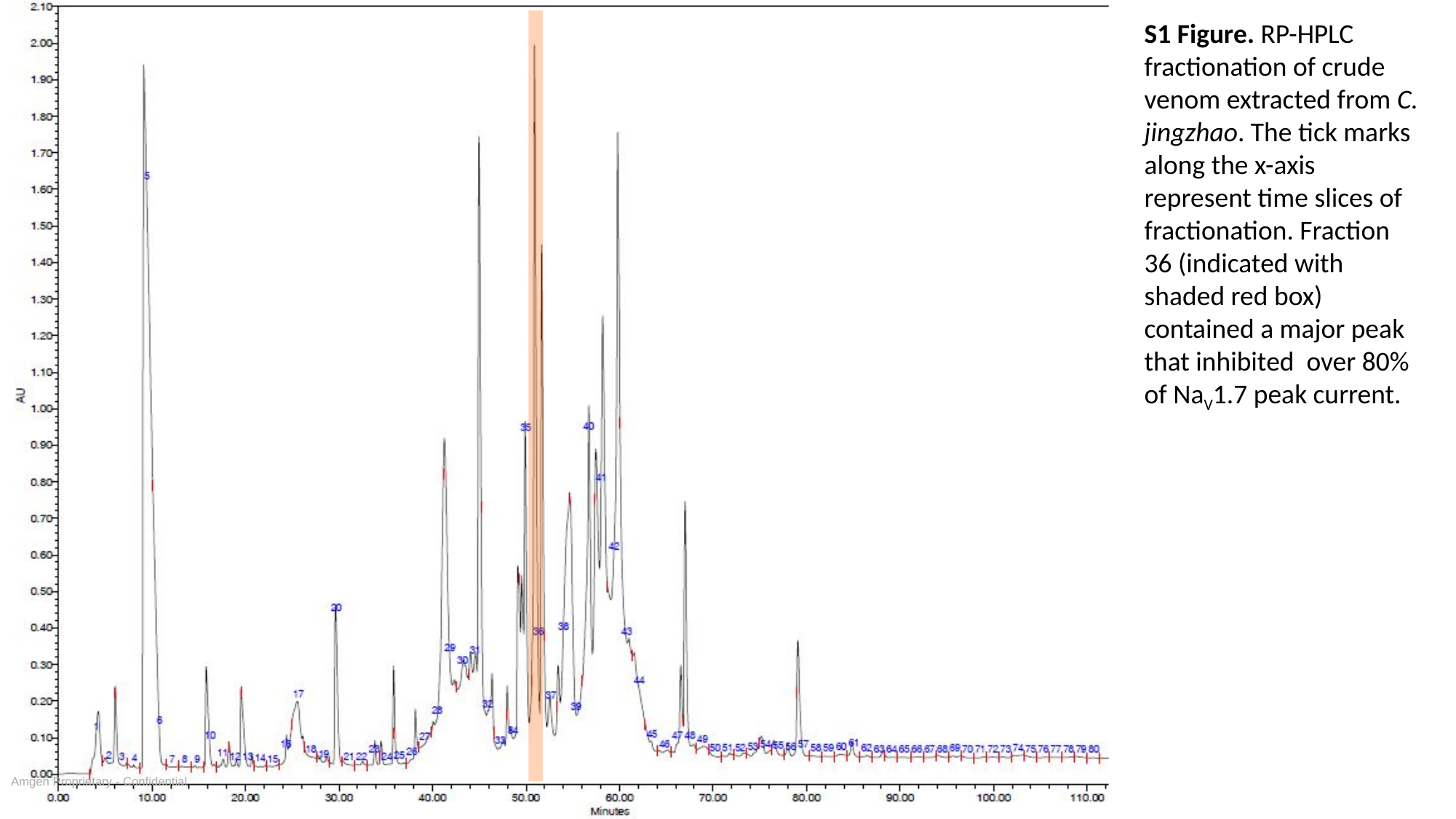

S1 Figure. RP-HPLC fractionation of crude venom extracted from C. jingzhao. The tick marks along the x-axis represent time slices of fractionation. Fraction 36 (indicated with shaded red box) contained a major peak that inhibited over 80% of NaV1.7 peak current.
Amgen Proprietary - Confidential
