# Supplementary material for: Pharmacological characterization of potent and selective NaV1.7 inhibitors engineered from Chilobrachys jingzhao tarantula venom peptide JzTx-V
Source: PLoS One. 2018 May 3;13(5):e0196791. doi: 10.1371/journal.pone.0196791 (PMC5933747; doi:10.1371/journal.pone.0196791)
Supplement: S2 Fig — (PPTX) [file pone.0196791.s003.pptx]

## Slide 1
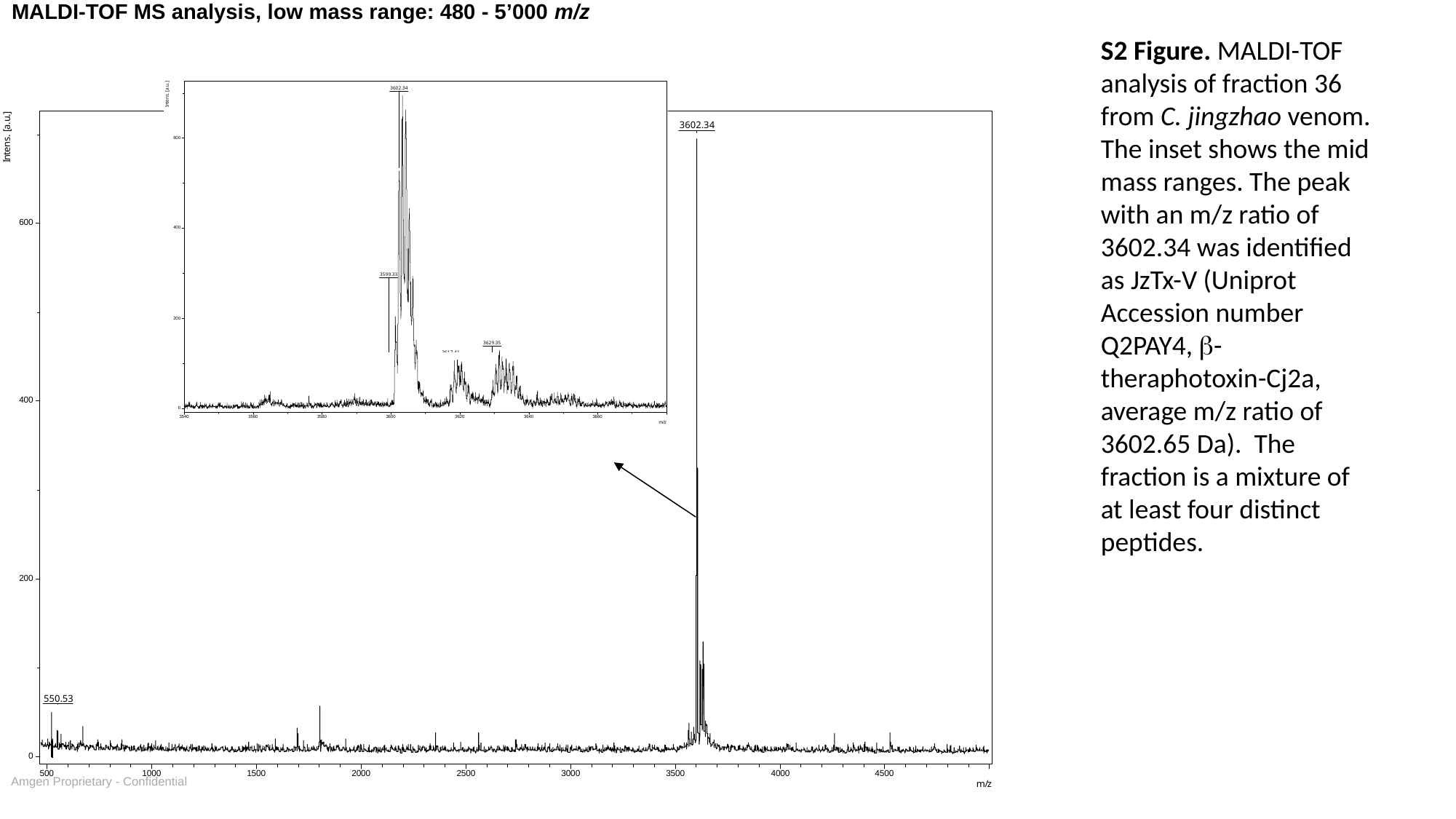

MALDI-TOF MS analysis, low mass range: 480 - 5’000 m/z
S2 Figure. MALDI-TOF analysis of fraction 36 from C. jingzhao venom. The inset shows the mid mass ranges. The peak with an m/z ratio of 3602.34 was identified as JzTx-V (Uniprot Accession number Q2PAY4, b-theraphotoxin-Cj2a, average m/z ratio of 3602.65 Da). The fraction is a mixture of at least four distinct peptides.
Amgen Proprietary - Confidential
