# Supplementary material for: Pharmacological characterization of potent and selective NaV1.7 inhibitors engineered from Chilobrachys jingzhao tarantula venom peptide JzTx-V
Source: PLoS One. 2018 May 3;13(5):e0196791. doi: 10.1371/journal.pone.0196791 (PMC5933747; doi:10.1371/journal.pone.0196791)
Supplement: S3 Fig — (PPTX) [file pone.0196791.s004.pptx]

## Slide 1
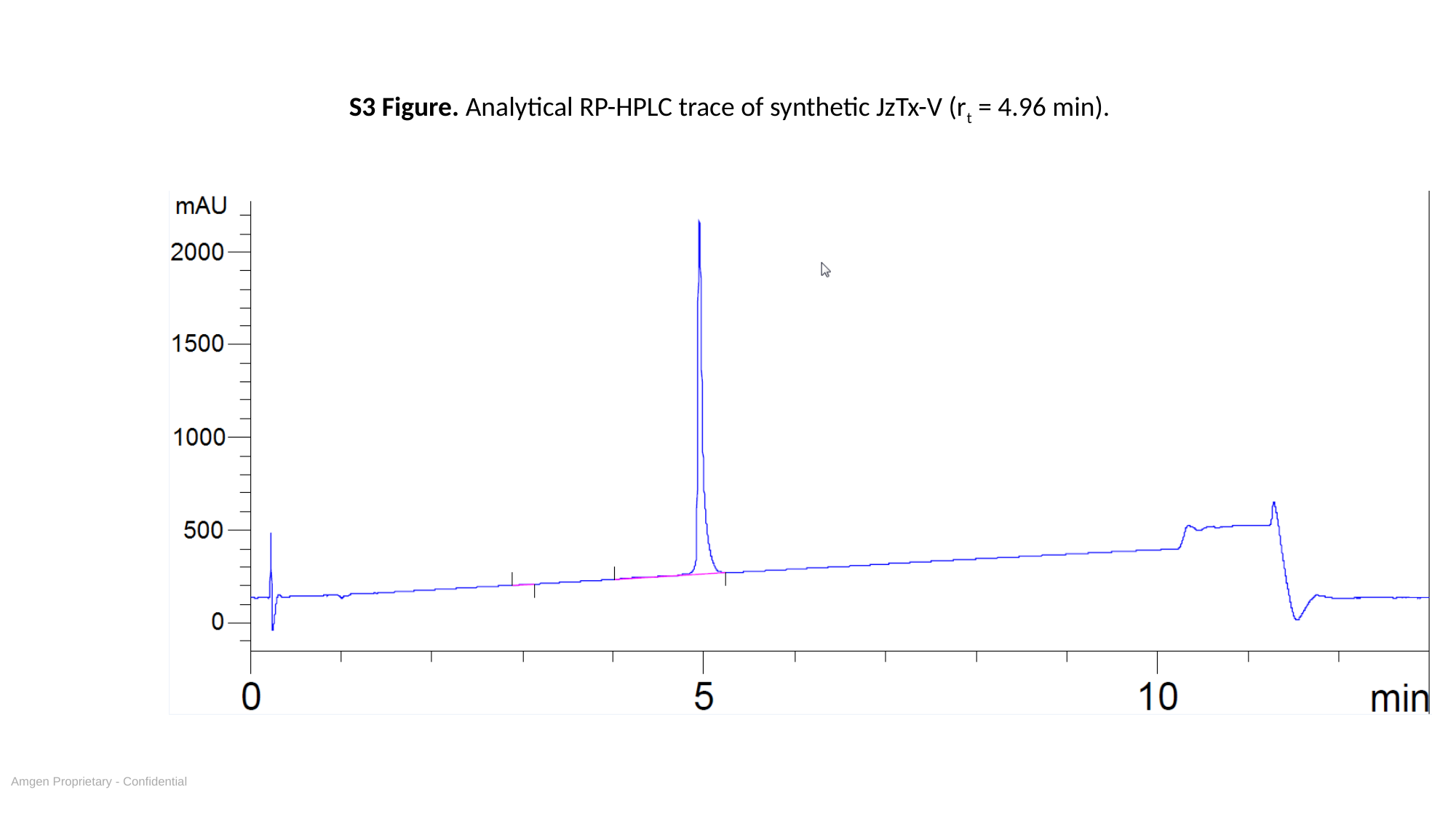

S3 Figure. Analytical RP-HPLC trace of synthetic JzTx-V (rt = 4.96 min).
Amgen Proprietary - Confidential
